# Knowledge on relative energy deficiency in sport among physiotherapists and physicians

**DOI:** 10.1002/ejsc.12026

**Published:** 2024-01-30

**Authors:** Saskia J. Verhoef, Merel C. Wielink, Edwin A. Achterberg, Marlies Y. Bongers, Simone M. T. A. Goossens

**Affiliations:** ^1^ Maastricht University Faculty of Health, Medicine and Life Sciences Maastricht the Netherlands; ^2^ Department of Obstetrics and Gynaecology Máxima Medical Centre Veldhoven the Netherlands; ^3^ Department of Sports Medicine Máxima Medical Centre Veldhoven the Netherlands; ^4^ Maastricht University Research School Grow Maastricht the Netherlands; ^5^ Eindhoven MedTech Innovation Center (e/MTIC) Eindhoven the Netherlands

**Keywords:** education, exercise, physiotherapy, weight

## Abstract

Relative energy deficiency in sport (RED‐S) refers to an impaired physiological functioning caused by low energy availability in both male and female athletes. It may affect many physiological processes, causing, among others, stress fractures and disturbed menstrual cycles. Athletes may present these symptoms to different health care workers. The purpose of this research was to investigate the awareness and knowledge of physiotherapists, general practitioners, gynaecologists, sports physicians and orthopaedic surgeons on RED‐S. An online questionnaire on awareness and knowledge of RED‐S was developed for the above‐mentioned health experts. A total number of 799 respondents were included for analysis, and 22.0% of the respondents had heard of the existence of RED‐S. The highest percentage was reported by sports physicians (92.9%) and the lowest among general practitioners (10.1%). Sports physicians scored highest on knowledge questions about RED‐S with a mean score of 8.9, and physiotherapists scored lowest with a mean score of 5.7. Of all respondents, 57.6% reported feeling competent discussing possible underlying problems of RED‐S and 7.4% felt qualified treating RED‐S. Of the responding gynaecologists, 46.6% would suggest starting oral contraception when presented a patient with symptoms of RED‐S, thereby only masking the symptoms of amenorrhoea but not treating the underlying cause: a low energy availability. This research reports a low awareness and knowledge among participating physiotherapists, general practitioners, gynaecologists and orthopaedic surgeons on RED‐S. Therefore, further education for these specialists on this topic is needed, since most athletes will initially consult these health care providers first.

## Highlights

- The great majority of all participating general practitioners are not aware of the existence of relative energy deficiency in sport (RED‐S).
- Awareness of RED‐S needs to be increased among at least physiotherapists, general practitioners, gynaecologists and orthopaedic surgeons to improve diagnosis and treatment of RED‐S.
- Sports physicians have the highest knowledge on RED‐S.

## 1 INTRODUCTION

Relative energy deficiency in sport (RED‐S) is a relatively new syndrome, as it was introduced in 2014 by the International Olympic Committee (IOC). RED‐S is caused by low energy availability, due to an imbalance of energy intake and exercising energy expenditure (Holtzman et al., 2019; Mountjoy et al., 2018). RED‐S includes health impairments, such as menstrual function, bone health, immunity and cardiovascular health and may cause, among others, stress fractures, disturbed menstrual cycles and psychological impairments (Mountjoy et al., 2014). The amount of research on RED‐S has increased significantly, but details about the consequences of low energy availability remain unclear (Logue et al., 2020).

RED‐S is an expansion of what was previously known as the Female Athlete Triad (Mountjoy et al., 2014). This triad was defined in 1992 as disordered eating, amenorrhoea and osteoporosis (Yeager et al., 1993). However, as additional symptoms and consequences were mentioned in athletes, including male athletes, RED‐S was thus introduced. Former research on the awareness and knowledge of the Female Athlete Triad focused mainly on coaches and trainers and described 64%–99% of all included coaches having heard of the Female Athlete Triad (Kroshus et al., 2018; Pantano, 2006). The awareness of paramedics and physicians on this topic was reported to be much lower. Depending on the setting, 28%–37% of physicians and nurses reported to have heard of the triad (Curry et al., 2015; Kroshus et al., 2015). This awareness was not examined separately for all specialists involved in recognition and treatment of the Female Athlete Triad, namely physiotherapists, general practitioners and sports physicians. Since these results date back to 2015, awareness might have increased since then.

Female athletes mention the (perceived) limited awareness and knowledge of general practitioners on menstrual problems among female athletes as one of the most important reasons not to seek help in case of amenorrhoea (Verhoef et al., 2021). Female athletes also report normalisation of absence of menstruation within their environment and embarrassment as reasons not to seek help in case of unexplained amenorrhoea (Chen et al., 2018). Sufficient knowledge on RED‐S among physiotherapists, general practitioners, orthopaedic surgeons and gynaecologists is important because they play an important role in signalling and diagnosing RED‐S. Especially general practitioners and physiotherapists, as primary care health care workers, are important since they are the first in line of health care professionals for most (amateur) athletes. Athletes may present with several symptoms associated with the syndrome, among others repetitive injuries, stress fractures or amenorrhoea. Sports physicians are often involved as a team doctor or may see patients with RED‐S who present with injuries or complaints such as unexplained tiredness. Orthopaedic surgeons might be confronted with patients suffering from stress fractures, while gynaecologists may see patients with RED‐S presenting with amenorrhoea or other menstrual cycle problems. Therefore, the purpose of this research was to investigate the awareness and knowledge of physiotherapists, general practitioners, gynaecologists, sports physicians and orthopaedic surgeons on RED‐S.

## 2 MATERIALS AND METHODS

An online questionnaire was developed for physiotherapists, general practitioners, gynaecologists, sports physicians, orthopaedic surgeons and residents of these specialties. Participants filled in the questionnaire between 12 October 2020 and 16 November 2020.

### 2.1 Development of questionnaire

Three of the authors (SJV, MCW and SMTAG) developed the questionnaire, and seven physiotherapists and physicians of the involved medical specialties (EAA, MYB and five others) reviewed the survey. The survey was adjusted based on their feedback. The average time to fill in the questionnaire was 5–10 min which was assessed as acceptable. Qualtrics survey software was used to program the questionnaire and send it to the target population.

### 2.2 Recruitment

A mass mail was sent to Dutch gynaecologists (*n* = 1498) through the NVOG, (Dutch association of gynaecologists) and sports physicians (*n* = 143) through the VSG (Dutch association of sports physicians). Since it was not possible to send a mass mail to physiotherapists, general practitioners and orthopaedic surgeons, they were contacted separately by mail. Physiotherapists were mainly reached through FysioVisie (Dutch network of 25 physiotherapy practices) with contact details found on ZorgkaartNederland (Dutch website of healthcare). General practitioners were contacted through details found on ZorgkaartNederland and by asking them to forward the survey to colleagues. Orthopaedic surgeons of three hospitals were personally approached and were asked to forward the survey to all their colleagues. Secretaries of orthopaedic departments of eight other hospitals were requested to forward the survey to the orthopaedic surgeons. Furthermore, social media accounts (LinkedIn and Instagram) of the authors were used to invite possible participants.

### 2.3 Components of questionnaire

After informed consent was obtained, the questionnaire was started.

**FIGURE 1:**
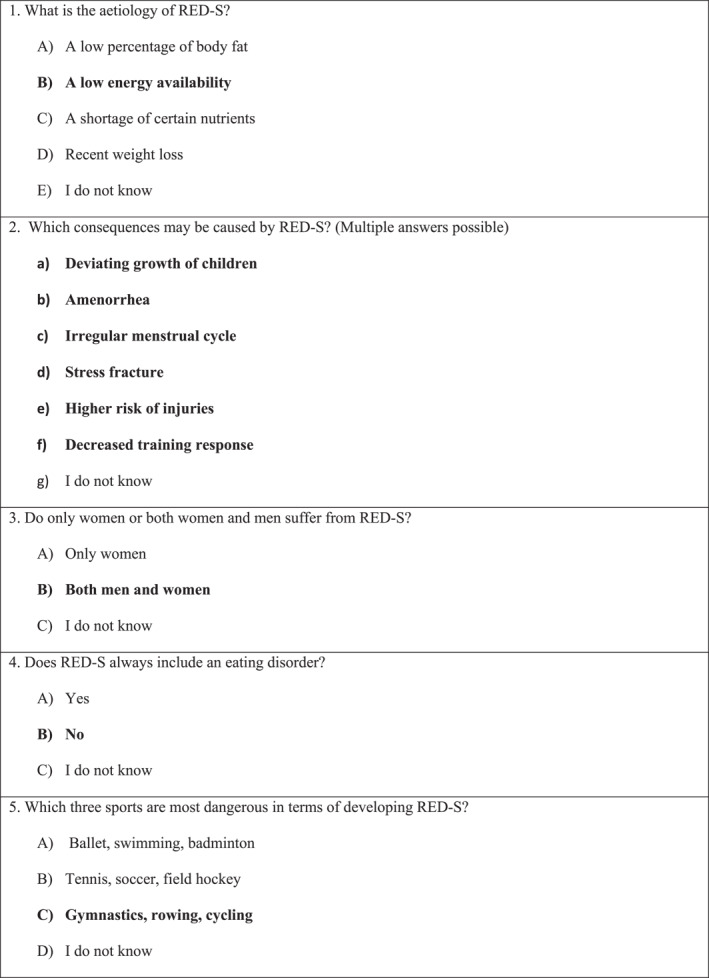
The five questions about aetiology, risks and consequences of relative energy deficiency in sport in the survey. Correct answers are in bold. Translated from Dutch in the original questionnaire.

The first part of the questionnaire consisted of general questions regarding baseline information, such as age and self‐reported affinity to sports. Subsequently, a fictional case was presented of a 23‐year‐old female with symptoms of RED‐S, depending on the medical specialty of the participant; the patient presented to a physiotherapist, sports physician or orthopaedic surgeon was diagnosed with a tibial stress fracture, and the patient who was presented to general practitioners and gynaecologists reported to have secondary amenorrhoea. The participant was then asked to specify what questions would seem relevant in their anamnesis (e.g. weight loss, [absence of] menstrual cycle, history of injuries or stress fractures) and what their subsequent treatment plan would be (e.g. a wait‐and‐see approach or referral to [another] medical specialist). These questions were multiple choice, with the possibility to add another answer in the ‘otherwise, namely’ option. Next, the participant was asked whether they are familiar with the term ‘RED‐S’. If so, the participant was asked to answer five questions regarding aetiology, risks and consequences of RED‐S (Figure 1). If the participant was not familiar with RED‐S, they proceeded to the next part of the questionnaire in which a short introduction on RED‐S was given. Thereafter, all participants were asked if they would feel competent discussing underlying problems of RED‐S (such as possible eating disorders) and if they would feel qualified to treat patients with RED‐S. Lastly, participants were asked who, according to their opinion, should be involved in the treatment of patients with RED‐S.

### 2.4 Analysis

Reasons for exclusion were: professions other than physiotherapist, general practitioner, gynaecologist, sports physician and orthopaedic surgeon not completing questionnaire for at least 50%, absence of informed consent and working outside of the Netherlands.

A descriptive analysis was performed to define characteristics of the responders and frequencies of answers given by the responders. Data analysis was performed using IBM SPSS Statistics V 25.0 and Excel. Primary outcomes were percentage of participants who were aware of the existence of the RED‐S syndrome and number of points on questions about knowledge of RED‐S (two points were given for each correct question). Continuous variables were presented as averages with 95% confidence intervals or interquartile ranges (IQR), depending on their distribution.

The Daily Board of the Medical Ethics Committee of Máxima Medical Centre confirmed that the rules laid down in the Medical Research Involving Human Subjects Act do not apply to this research (METC‐number N20.099).

## 3 RESULTS

Response rates were 19.2% for gynaecologists and 63.64% for sports physicians. An estimation of response rates for physiotherapists, general practitioners and orthopaedic surgeons was not possible due to the nature of the dissemination of the questionnaire.

After exclusion, a total number of 799 responses were included for analysis. In total, 83 responses were excluded because of reported profession other than the abovementioned professions (*n* = 18, e.g. physician assistant *n* = 4, dietitian *n* = 1 and midwife *n* = 1), not completing questionnaire for at least 50% (*n* = 56), absence of informed consent (*n* = 8) and working outside of the Netherlands (*n* = 1). Baseline characteristics of the different health care providers are reported in Table 1.

**TABLE 1:** Baseline characteristics of responders.

	Physiotherapists (*n* = 189)	General practitioners (*n* = 200)	Gynaecologists (*n* = 281)	Orthopaedic surgeons (*n* = 43)	Sports physicians (*n* = 86)
Gender % (*n*)					
Male	37.0 (70)	26.5 (53)	16.4 (46)	69.8 (30)	47.7 (41)
Female	63.0 (119)	73.5 (147)	83.6 (235)	30.2 (13)	52.3 (45)
Age % (*n*)					
≤30 years	37.0 (70)	23.5 (47)	11.0 (31)	14.0 (6)	12.8 (11)
31–40 years	33.3 (63)	39.0 (78)	42.3 (119)	34.9 (15)	46.5 (40)
41–50 years	9.5 (18)	16.0 (32)	27.8 (78)	30.2 (13)	18.6 (16)
51–60 years	14.3 (27)	16.0 (32)	14.9 (42)	14.0 (6)	18.6 (16)
≥61 years	5.8 (11)	5.5 (11)	3.9 (11)	7.0 (3)	3.5 (3)
Average amount of hours of weekly sport practice per week in preceding year % (*n*)					
<2 h	21.2 (40)	34.5 (69)	44.8 (126)	20.9 (9)	17.4 (15)
≥2, <4 h	36.0 (68)	44.5 (89)	35.2 (99)	37.2 (16)	43.0 (37)
≥4, <6 h	24.3 (46)	15.5 (31)	13.9 (39)	25.6 (11)	22.1 (19)
≥6, <8 h	9.5 (18)	4.0 (8)	3.2 (9)	4.7 (2)	14.0 (12)
≥8, <10 h	5.8 (11)	0.5 (1)	1.8 (5)	7.0 (3)	2.3 (2)
≥10 h	3.2 (6)	1.0 (2)	1.1 (3)	4.7 (2)	1.2 (1)
Highest level of sport competition in lifetime % (*n*)					
Recreative	33.3 (63)	46.0 (92)	44.8 (126)	41.9 (18)	22.1 (19)
Competitive	52.4 (99)	47.5 (95)	51.2 (144)	53.5 (23)	55.8 (48)
(Semi)professional	14.3 (27)	6.5 (13)	3.9 (11)	4.7 (2)	22.1 (19)
Self‐reported affinity to sports (0–10) mean (95% CI)	8.2 (8.0–8.4)	7.1 (6.9–7.3)	6.7 (6.5–7.0)	7.7 (7.2–8.1)	8.8 (8.6–9.0)
Post‐residency experience % (*n*)					
Still in residency	1.6 (3)	28.0 (56)	31.3 (88)	23.3 (10)	26.7 (23)
≤5 years	24.9 (47)	27.5 (55)	19.9 (56)	18.6 (8)	18.6 (16)
6–10 years	27.0 (51)	13.5 (27)	17.1 (48)	14.0 (6)	22.1 (19)
11–15 years	12.7 (24)	8.5 (17)	13.9 (39)	20.9 (9)	11.6 (10)
16–20 years	9.0 (17)	6.5 (13)	6.8 (19)	9.3 (4)	7.0 (6)
21–25 years	6.9 (13)	6.5 (13)	6.8 (19)	4.7 (2)	11.6 (10)
26–30 years	6.3 (12)	5.0 (10)	3.2 (9)	9.3 (4)	0.0 (0)
≥31 years	11.6 (22)	4.5 (9)	1.1 (3)	0.0 (0)	2.3 (2)

### 3.1 Anamnesis, referral policy and suggested treatment

Physiotherapists, sports physicians and orthopaedic surgeons were presented with a case of a 23‐year‐old female patient with a normal BMI who trained 15 h per week for a triathlon who presented with a stress fracture. Almost all responding physiotherapists (96.2%, *n* = 177), all responding sports physicians (*n* = 86) and 93% (*n* = 40) of the responding orthopaedic surgeons would ask for a recent increase of sport intensity. Almost half (47.3%, *n* = 87) of the responding physiotherapists, the majority of the responding sports physicians (86%, *n* = 74) and only 18.6% (*n* = 8) of the responding orthopaedic surgeons would also ask for weight loss. Almost 20% (18.5%, *n* = 34) of the responding physiotherapists, 89.5% (*n* = 77) of the responding sports physicians and 11.6% (*n* = 5) of the responding orthopaedic surgeons would ask questions regarding menstrual cycle.

Subsequently, the responding physiotherapists, sports physicians and orthopaedic surgeons were asked what their next steps would be in case the patient reported an absence of menstruation in the last 8 months with a stable body weight. Most responding physiotherapists (83.7%, *n* = 154) indicated they would send a report to the general practitioner and advise the patient to contact their general practitioner. Of all responding sports physicians, 61.6% (*n* = 53) indicated that they would advise to stop or decrease in the intensity of sport, 57.0% (*n* = 49) would refer to a (sports) dietitian and 25.6% (*n* = 22) would refer to a gynaecologist. For orthopaedic surgeons, referral to a sports physician (44.2%, *n* = 19) and referral to a gynaecologist (37.2%, *n* = 16) were the most chosen options.

Both general practitioners and gynaecologists were presented a case of a 23‐year‐old female patient with a normal BMI and amenorrhoea in the last 8 months. Of the responding general practitioners, 74% (*n* = 148) would ask about (an increase in) intensity of sports activities and 85.4% (*n* = 239) of the gynaecologist reported to do so. A minority of both responding general practitioners (5%, *n* = 10) as well as gynaecologists (6.4%, *n* = 18) would ask for a history of injuries or stress fractures. Most general practitioners (57%, *n* = 114) suggested a wait‐and‐see approach and would reassure the patient that the menstrual cycle would recur when the patient would decrease the intensity of sports, but with no need to immediately do so. The most chosen treatment plan by the responding gynaecologists was the prescription of the contraceptive pill to regulate the menstrual cycle (46.6%, *n* = 131), followed by a wait‐and‐see approach by 42.0% (*n* = 118). Multiple responders mentioned starting a contraceptive pill for hormone suppletion and not for regulating the menstrual cycle.

### 3.2 Knowledge and feeling competent

In total, 22.0% (*n* = 175) of the respondents were aware of the existence of the RED‐S syndrome. This percentage was highest among the responding sports physicians, of whom 92.9% (*n* = 79) reported being aware of the existence of the RED‐S syndrome, and the lowest among the responding general practitioners, of whom only 10.1% (*n* = 20) reported being aware of this syndrome. Of the responding orthopaedic surgeons, 23.3% (*n* = 10) were aware of the existence of the RED‐S syndrome, 18.5% (*n* = 35) of the responding physiotherapists and 11.0% (*n* = 31) of the responding gynaecologists.

**FIGURE 2:**
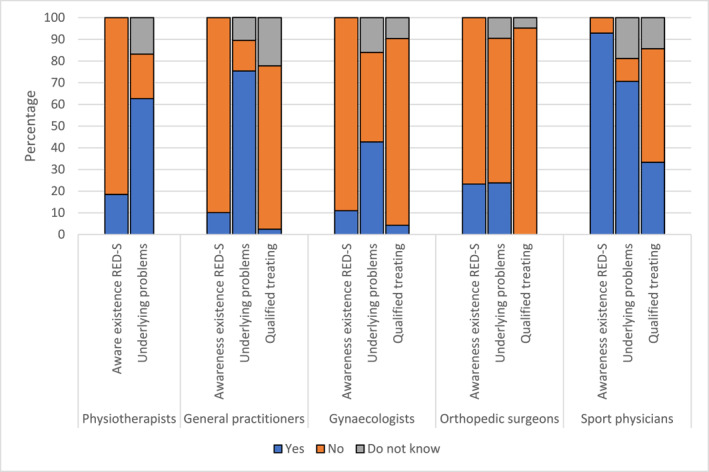
Awareness of existence of relative energy deficiency in sport (RED‐S), feeling qualified discussing underlying problems and feeling qualified treating RED‐S among the different specialists.

Of the respondents answering this question, 57.6% (*n* = 456) answered feeling competent discussing possible underlying problems of RED‐S, 27.7% (*n* = 219) reported they do not feel qualified to do this and 14.8% (*n* = 117) reported they do not know. Of the respondents who answered the question, 7.4% (*n* = 45) felt qualified treating RED‐S. In Figure 2, the awareness and feeling qualified to discuss underlying problems and treat RED‐S are presented for the different specialists.

The mean score of the questions about RED‐S (*n* = 175) was 7.7 (IQR 7–10). Physiotherapists scored lowest on these questions with a mean score of 5.7 (IQR 4–7), followed by the orthopaedic surgeons (mean 6.1, IQR 3–8.25), general practitioners (mean 7.3, IQR 5.5–9) and gynaecologists (mean 7.8, IQR 7–9). The highest mean score of 8.9 (IQR 9–10) was achieved by the sports physicians.

According to the respondents, sports physicians (68.8%, *n* = 539), followed by (sports) dietitians (63.9%, *n* = 501), (sports) psychologists (49.6%, *n* = 389), (sports) physiotherapists (29.3%, *n* = 230) and general practitioners (18.1%, *n* = 142) are the specialists who should particularly be involved in the treatment of RED‐S. A discrepancy was seen between the desired involvement of (sports) physiotherapists in the treatment of RED‐S answered by the medical specialty of the responder. Of all responding sports physicians, 4.8% felt physiotherapists should be involved in treating RED‐S as compared to 43.5% of all physiotherapists who felt physiotherapists should be involved. Most responders (76.4%, *n* = 599) chose more than one specialist and multiple responders added ‘multidisciplinary’ in the comments.

## 4 DISCUSSION

Overall, awareness of the existence of RED‐S is low with an average percentage of only 22% of the respondents heaving heard of this syndrome, suggesting education on RED‐S is needed. Responding sports physicians had a relatively high percentage (92.9%) of knowledge of the existence of RED‐S compared to responding physiotherapists, general practitioners, gynaecologists and orthopaedic surgeons. Furthermore, responding sports physicians scored highest on the questions about RED‐S. Therefore, education on RED‐S should mainly focus on physiotherapists, general practitioners, gynaecologists and orthopaedic surgeons.

Striking differences were noted in the answers among the different specialists with respect to feeling qualified to discuss underlying problems and feeling qualified to treat RED‐S. Especially among general practitioners, a big difference between these two questions was noted; although 75.4% of the general practitioners reported feeling competent discussing underlying problems, only 2.5% reported feeling competent to treat RED‐S. General practitioners are skilled at starting a conversation with a patient and thereby identifying underlying problems. In addition, they are well trained in assessing whether they can provide the treatment themselves or should refer. However, it is clear that most general practitioners would not feel competent treating RED‐S themselves.

Remarkably, 46.6% of the responding gynaecologists would suggest starting oral contraception when presented with a patient with amenorrhoea who trains 15 h a week. Starting oral contraception would only mask the amenorrhoea but does not treat the underlying problem of low energy availability and the associated problems such as decreased bone health. The bone loss may progress or even worsen with this treatment, especially if the athlete maintains low energy availability (Gordon et al., 2017; Hartard et al., 2007). It is thus very important to educate gynaecologists and general practitioners who treat these patients on the topic and the preferred treatment methods.

Although we have tried to reach a representative group of respondents of each specialty as possible, selection bias cannot be ruled out. Especially, physicians who had little knowledge on menstrual problems and/or sports may have been less inclined to fill in the questionnaire with the subject ‘menstrual problems with athletes’. Also, the majority of the responders were female. Although most physiotherapists and physicians are female in the Netherlands (58.8% and 56.6%), the percentage of female responders in the questionnaire is even higher (72.1% of the physicians and 63% of the physiotherapists) (Ministerie van Volksgezondheid, 2021) This selection bias may have resulted in an overestimation of both awareness and knowledge on RED‐S as compared to the general population of these specialists, and an overestimation of physicians who feel competent discussing underlying problems and feeling qualified treating RED‐S. Most responders answered more than one specialist should be involved in the treatment of RED‐S, and multiple responders added ‘multidisciplinary’ in the comments, indicating a multidisciplinary team is needed for the treatment of RED‐S. This is in line with the healthcare professional recommendations on RED‐S of the IOC, in which a multidisciplinary team is recommended including a sports physician, nutritionist, psychologist, physiotherapist and physiologist (Mountjoy et al., 2014). The five specialists chosen most in the questionnaire were sports physicians, (sports) dietitians, (sports) psychologists, (sports) physiotherapists and general practitioners. The (sports) physiotherapists were chosen most by the (sports) physiotherapists themselves and less by the other responders.

The questionnaire was conducted in the Netherlands and therefore represents the Dutch situation. It is questionable if this research also reflects the current knowledge in other (European) countries. In the USA, an awareness of 37% was reported on the Female Athlete Triad among physicians (Curry et al., 2015). However, this cannot be compared to our awareness percentage of 22% on RED‐S among physicians, since our research is about awareness on RED‐S and not on the Female Athlete Triad. As far as we know, our survey has been the first research in Europe that examines the knowledge of physicians on RED‐S. This research may inspire researchers in other (European) countries to perform similar research. Results of these surveys in other countries will clarify if the low awareness and knowledge is a problem unique for the Netherlands or also an international issue.

In the questionnaire, the participants were asked if they ever heard of RED‐S and not if they know the Female Athlete Triad. Therefore, it might be possible that a significant amount of participants who filled in they never heard of RED‐S before, did have knowledge on the Female Athlete Triad. Furthermore, paediatricians and nutritionists were not surveyed in this research because in former research they were not mentioned by athletes as one of the physicians or paramedics they would go to when experiencing menstrual disorders (Verhoef et al., 2021). Since knowledge on RED‐S and/or a low energy availability is also important for them, this might be an item for future research.

We belief one of the solutions of the lack of knowledge is more education for gynaecologists on RED‐S. Máxima MC started organising schoolings for gynaecologists on RED‐S. Furthermore, we have set up a network of sports and gynaecology where athletes can refer to with questions related to menstrual cycle (disturbances). Through this network, they will be contacted with gynaecologists or sports physicians with specific knowledge on the subject.

## 5 CONCLUSION

Multiple paramedics and specialists play an important role in the recognition and treatment of RED‐S, including physiotherapists, general practitioners, gynaecologists, orthopaedic surgeons and sports physicians. We report a low awareness among responding physiotherapists, general practitioners, gynaecologists and orthopaedic surgeons on RED‐S. Furthermore, many physicians suggested wrong treatment options by suggesting either a wait‐and‐see approach or starting oral contraception. For those who were familiar with RED‐S, sports physicians scored highest on questions about knowledge of RED‐S but most sports physicians reported not feeling qualified to treat RED‐S (on their own). The low awareness indicates further education for these specialists on this topic is needed, by for example, integrating education about RED‐S in the curricula for medical students or organizing refresher courses for (residents of) the involved specialties.

## AUTHOR CONTRIBUTIONS

**Saskia J. Verhoef**: contribution to study conception, design, material preparation, data collection, analysis, writing first draft of manuscript. **Merel C. Wielink**: contribution to study conception, design, material preparation, data collection, analysis, writing of article. **Edwin A. Achterberg**: material preparation, critical review of article. **Marlies Y. Bongers**: contribution to study conception, design, material preparation, critical review of article. **Simone M. T. A. Goossens**: contribution to study conception, design, material preparation, data collection, analysis, writing of article. All authors commented on previous versions of the manuscript. All authors read and approved the final manuscript.

## CONFLICT OF INTEREST STATEMENT

The authors declare that they have no competing interests.

## Data Availability

Data are stored on a protected disk of computers in Máxima Medical Centre. The datasets used and analysed during the current study are available from the corresponding author on reasonable request.

## References

[ejsc12026-bib-0001] Chen, Chen X. , Carol Shieh , Claire B. Draucker , and Janet S. Carpenter . 2018. “Reasons Women Do Not Seek Health Care for Dysmenorrhea.” Journal of Clinical Nursing 27(1–2): e301–e308. 10.1111/jocn.13946. 28681499 PMC5746430

[ejsc12026-bib-0002] Curry, Emily J. , Catherine Logan , Kathryn Ackerman , Kelly C. McInnis , and Elizabeth G. Matzkin . 2015. “Female Athlete Triad Awareness Among Multispecialty Physicians.” Sports Medicine Open 1(1): 38. 10.1186/s40798-015-0037-5. 26587370 PMC4642583

[ejsc12026-bib-0003] Gordon, Catherine M. , Kathryn E. Ackerman , Sarah L. Berga , Jay R. Kaplan , George Mastorakos , Madhusmita Misra , Mohammad Hassan Murad , Nanette F. Santoro , and Michelle P. Warren . 2017. “Functional Hypothalamic Amenorrhea: An Endocrine Society Clinical Practice Guideline.” Journal of Clinical Endocrinology & Metabolism 102(5): 1413–1439. 10.1210/jc.2017-00131. 28368518

[ejsc12026-bib-0004] Hartard, Manfred , Christine Kleinmond , Michael Wiseman , Ernst R. Weissenbacher , Dieter Felsenberg , and Reinhold G. Erben . 2007. “Detrimental Effect of Oral Contraceptives on Parameters of Bone Mass and Geometry in a Cohort of 248 Young Women.” Bone 40(2): 444–450. 10.1016/j.bone.2006.08.001. 16965947

[ejsc12026-bib-0005] Holtzman, Bryan , and Kathryn E. Ackerman . 2019. “Measurement, Determinants, and Implications of Energy Intake in Athletes.” Nutrients 11(3): 665. 10.3390/nu11030665. 30893893 PMC6472042

[ejsc12026-bib-0006] Kroshus, Emily , Anastasia N. Fischer , and Jeanne F. Nichols . 2015. “Assessing the Awareness and Behaviors of U.S. High School Nurses with Respect to the Female Athlete Triad.” The Journal of School Nursing 31(4): 272–279. 10.1177/1059840514563760. 25530174

[ejsc12026-bib-0007] Kroshus, Emily , J. D. DeFreese , and Zachary Y. Kerr . 2018. “Collegiate Athletic Trainers' Knowledge of the Female Athlete Triad and Relative Energy Deficiency in Sport.” Journal of Athletic Training 53(1): 51–59. 10.4085/1062-6050-52.11.29. 29251536 PMC5800729

[ejsc12026-bib-0008] Logue, Danielle M. , Sharon M. Madigan , Anna Melin , Eamonn Delahunt , Mirjam Heinen , Sarah‐Jane Mc Donnell , and Clare A. Corish . 2020. “Low Energy Availability in Athletes 2020: An Updated Narrative Review of Prevalence, Risk, Within‐Day Energy Balance, Knowledge, and Impact on Sports Performance.” Nutrients 12(3): 835. 10.3390/nu12030835. 32245088 PMC7146210

[ejsc12026-bib-0009] Ministerie van Volksgezondheid, Welzijn en Sport: BIG‐Register. 2021. Accessed October 31, 2021. https://www.bigregister.nl/over‐het‐big‐register/cijfers.

[ejsc12026-bib-0010] Mountjoy, Margo , Jorunn Kaiander Sundgot‐Borgen , Louise M. Burke , Kathryn E. Ackerman , Cheri Blauwet , Naama Constantini , Constance Lebrun , et al. 2018. “IOC Consensus Statement on Relative Energy Deficiency in Sport (RED‐S): 2018 Update.” British Journal of Sports Medicine 52(11): 687–697. 10.1136/bjsports-2018-099193. 29773536

[ejsc12026-bib-0011] Mountjoy, Margo , Jorunn Sundgot‐Borgen , Louise Burke , Susan Carter , Naama Constantini , Constance Lebrun , Nanna Meyer , et al. 2014. “The IOC Consensus Statement: Beyond the Female Athlete Triad–Relative Energy Deficiency in Sport (RED‐S).” British Journal of Sports Medicine 48(7): 491–497. 10.1136/bjsports-2014-093502. 24620037

[ejsc12026-bib-0012] Pantano, Kathleen J. 2006. “Current Knowledge, Perceptions, and Interventions Used by Collegiate Coaches in the U.S. Regarding the Prevention and Treatment of the Female Athlete Triad.” North American Journal of Sports Physical Therapy 1(4): 195–207. 21522222 PMC2953355

[ejsc12026-bib-0013] Verhoef, Saskia J. , Merel C. Wielink , Edwin A. Achterberg , Marlies Y. Bongers , and Simone M. T. A. Goossens . 2021. “Absence of Menstruation in Female Athletes: Why They Do Not Seek Help.” BMC Sports Science, Medicine & Rehabilitation 13(1): 146. 10.1186/s13102-021-00372-3. PMC8609260 34814941

[ejsc12026-bib-0014] Yeager, Kimberly K. , Rosemary Agostini , Aurelia Nattiv , and Barbara Drinkwater . 1993. “The Female Athlete Triad: Disordered Eating, Amenorrhea, Osteoporosis.” Medicine & Science in Sports & Exercise 25(7): 775–777. 10.1249/00005768-199307000-00003. 8350697

